# Microplastics: the hidden danger

**DOI:** 10.1016/j.jped.2024.10.004

**Published:** 2024-11-14

**Authors:** Marilyn Urrutia-Pereira, Paulo Augusto Camargos, Dirceu Solé

**Affiliations:** a Universidade Federal do Pampa (Unipampa), Faculdade de Medicina, Uruguaiana, RS, Brazil; b Universidade Federal de Minas Gerais (UFMG), Departamento de Pediatria, Belo Horizonte, MG, Brazil; c Universidade Federal de São Paulo (UNIFESP), Escola Paulista de Medicina (EPM), Departamento de Pediatria, Disciplina de Alergia, Imunologia Clínica e Reumatologia, São Paulo, SP, Brazil

**Keywords:** Microplastics, Nanoplastics, Human health, Toxicology, Marine litter

## Abstract

**Objective:**

To assess the impact of microplastics/nanoplastics (MiP/NP) on human health.

**Data source:**

The authors conducted a narrative review of articles published in English, Portuguese, French and Spanish in the last decade in the following databases: PubMed, Google Scholar, EMBASE and SciELO. The keywords used in this search were: microplastics OR nanoplastics OR marine litter OR toxicology OR additives AND human health OR children OR adults.

**Data synthesis:**

MiP is a group of emerging contaminants that have attracted increasing scientific interest and attention from society in the last decade due to their ubiquitous detection in all environments. Humans can be mainly exposed to MiP and NP orally, by inhalation, by dermal contact, as well as through systemic routes and cannot be neglected, especially in young children. The possible toxic effects in different systems are due to plastic particles, often combined with leachable additives and adsorbed contaminants.

**Conclusions:**

Unless the plastics value chain is transformed in the next two decades, the risks to species, marine ecosystems, climate, health, economies and communities will become unmanageable. However, alongside these risks lie unique opportunities to lead the transition to a more sustainable world.

## Introduction

While plastics have been heralded as a blessing for humanity, they also pose a hidden threat to human and planetary health.^1^ They bring enormous benefits, but the current patterns of production, use and disposal pay little attention to a sustainable design or the use of safe compounds, and the near absence of recovery, reuse and recycling are responsible for serious health problems, widespread environmental damage, large economic costs and profound social injustices.^1^

Although there are still gaps in the knowledge about the damage caused by plastics and uncertainties about their full magnitude, the currently available evidence clearly shows that these impacts are large and will increase in severity in the absence of urgent and effective intervention on a global scale.^1^

Plastic is a synthetic material widely used by humans due to its cost-effectiveness, durability, low weight, and the fact that it is easily manufactured.^2^

According to their diameter, plastics are characterized as: nanoplastics (< 1 mm; NP), microplastics (MiP, between 1 mm and 5 mm), mesoplastics (between 5 mm and 20 mm), macroplastics (> 20 mm) and megaplastics (larger than 100 mm).^3^

MiP can have different forms (fibers, fragments, spheres, granules, films, flakes, pellets and foam) depending on the plastic of origin format, the deterioration processes that occur on the surface of the plastic and the time of permanence in the environment.^3^ The potential factors that can affect the crossing of barriers and the effects of NP/MiP on health include characteristics of this material, exposure doses, administration routes, duration of exposure, co-exposure to other pollutants, and genetic predisposition.^4^

Among the plastic polymers found in MiP and NP particles, the following stand out: polyester (polycyclohexylenedimethylene terephthalate [PCT]), polypropylene (PP); polyvinyl chloride (PVC), polystyrene (PS), Teflon, nylon 6.6, polyethylene (PE), polyethylene terephthalate (PET), styrene-acrylonitrile resin (SAN) and poly(n‑butyl methacrylate) (PBMA).^5^

## Sources of microplastics

### Indoor environment

There are several direct and indirect sources of plastics in the indoor environment. These include personal care and use products, paints, synthetic grass in sports halls, abrasion of floors, furniture and textiles, and 3D printers.^6^

Soltani et al. evaluated the presence of MiP in Australian homes and demonstrated that the greatest risk of exposure to them occurred in children under six months, data confirmed by other authors.^7^ PE, PS, PA and polyacrylic (PAC) fibers were found in homes with carpets and PVC fibers in floors without carpets.^8^

During the laundry process, numerous fibers from natural or synthetic fabrics are released into the effluent (PS, PAC). These fibers are discharged into domestic sewage systems and sent to sewage treatment plants. In locations where there is no sewage collection or treatment network, the MiP input will have a major impact on the environment.^8^

### External environment

The concentrations of MiP in road dust are directly related to the volume of vehicle traffic, suggesting a relationship between the two.^9^ Tire wear particles (TWPs) are one of the most important sources of MiP worldwide. They consist of styrene butadiene rubber, in addition to the wear of road coatings, vehicle abrasion and weathering.^9^

The mechanisms of transport (ambient wind flow), dispersion (turbulence/local disturbance) and deposition (downward air movement) are responsible for the movement of MiP, which is aided by their size, length and shape.^10^

Koutnik et al. observed MiP concentrations in playgrounds that were on average five times higher than concentrations in other locations, outside the playground. The mean concentration of MiP in sand samples collected from the playground was higher and represented by more than 50 % polyethylene or polypropylene.^11^

MiP also serves as a substrate for organisms, which is called plastisphere, (plastisphere: communities that have evolved to live in man-made plastic environments, including fungi, bacteria, algae and viruses) and can act as dispersers of these organisms to other environments, with consequences for global health.^10^

MiP also interacts with pesticides, persistent organic pollutants, and heavy metals and act as a vector for the transfer of contaminants, such as pollen, endotoxins, and viruses in different environments and in the transport of microorganisms, including pathogens, by the formation of a biofilm on the surface of MiP^12^ that contributes to the worsening of asthma symptoms^13^ in addition to significantly increasing the efficiency of transfer of antibiotic resistance genes (ARGs).^14^

## Exposure routes

MiP pollution in ecosystems is widespread as a result of human activities, which makes human exposure to them inevitable and can occur through ingestion, inhalation, skin contact or systemic route.^11^ Table 1 lists the main routes of exposure to MIP.

**Table 1:** Routes of exposure to microplastics.^15^

*Digestive*
Main route of exposure associated with the ingestion of food contaminated with plastics (water from plastic bottles and/or contaminated with plastics, salt, shellfish and fish, among others).
*Respiratory*
Important source of exposure from particles released from automobile tires, synthetic clothing fibers, and other sources that are resuspended from surfaces such as walls or furniture, inhalation and/or nebulization devices.
*Cutaneous*
Small particles. Change in the epithelial barrier.
*Systemic (intravascular)*
Solutions used for infusion, parenteral nutrition, syringes, devices for installing venous access, cannulas, catheters or during the collection of biological material for analysis.

As an emerging type of pollutant, MiP is easily ingested by several organisms. Constant exposure to the open air increases the risk of fragmentation and mixing in the atmosphere, enhancing their deleterious action on biological functions due to their small size, high specific surface area, and strong biological penetration capacity.^16^^,^^17^

MiP has generated considerable concerns about potential risks to children's health, resulting from specific behaviors, especially during critical periods of immunological, respiratory, cardiological, and metabolic development, among others, which make the early years essential to avoid lasting damage to health.^18^

## Immune system

MiP can have an effect on the immune system due to their physicochemical characteristics. The bioaccumulation of MiP can disrupt metabolic balance and consequently disrupt the immune system efficiency. Once MiP are introduced into the human body, they can aggregate and exert localized toxicity by activating or enhancing immune responses, decreasing defense mechanisms against infections, and affecting the use of energy storage.^22^

Two major classes of chemicals related to plastics are of great concern to human health: bisphenols and phthalates. The administration of these chemicals during critical stages of development affects important components of the immune system and immune function, which may be related to the development of different diseases, including cancer.^23^

## Thyroid

Environmental exposure to bisphenol A and phthalates, which are very prevalent in plastics and personal care products, has been correlated with fluctuations in thyroid hormone levels, disturbances in the regulation of thyroid-stimulating hormone (TSH), and impaired development of the thyroid gland. Bisphenols can modify the thyroid hormone action by antagonizing the thyroid receptor action, altering gene expression, and mimicking thyroid transport proteins.^24^

## Respiratory system

Upon reaching the air-water interface in the alveolus, MiP/NP form vesicles that cause biophysical dysfunction of the surfactant, disrupting its structure and mobility and altering surface tension, determining its collapse to a greater or lesser extent and intensity.^25^ The uptake of NP/MiP by alveolar macrophages or lung epithelial cells is largely dependent on the size and type of material involved and can induce pro-inflammatory and oxidative stress responses.^26^

MiP can also affect nasal microecosystems since high exposure can increase the abundance of nasal microbiota associated with respiratory tract diseases, such as *Klebsiella sp* and *Helicobacter sp*, and reduce the abundance of beneficial ones such as Bacteroides.^27^

Studies reinforce the importance of the presence of MiP in cigarettes. A new form of cigarette pollution is added to the environment with proven damage to ecosystems. Cigarette filters comprise more than 15,000 fiber strands that can be detached into a range of sizes (microfibers/MiP) or eventually fragmented into smaller sizes.^28^

## Circulatory system

The intestinal vascular barrier may be compromised, allowing NP/MiP to enter the circulatory system and access the liver via the portal vein; thus, a substantial number of these aggregated protein-plastic complexes reach the circulatory system. They can cause artery obstruction and adhere to red blood cells in high proportion, resulting in red blood cell damage caused by mechanical, osmotic and oxidative stresses.^22^

## Reproductive system

Studies involving terrestrial mammals reveal that NP/MiP can induce reproductive toxicity through several mechanisms, such as reproductive toxicity that manifests predominantly as disruption of the blood-testicular barrier, impaired spermatogenesis, sperm malformation, sperm DNA damage, reduced sperm fertilization capacity, impaired oocyte maturation, impaired follicular growth, granulosa cell apoptosis, decreased ovarian reserve function, uterine and ovarian fibrosis, and endocrine disruption, among other effects.^29^

An experimental study with pregnant rats documented the presence of NP in their lungs, heart, and spleen. The same occurred with regard to the placenta and fetal tissues such as the liver, lungs, heart, kidney, and brain. These facts suggest that the fetoplacental unit is vulnerable to these aggressions that affect the fetus.^30^ In human placentas (fetal side, maternal side and chorioamniotic membrane) the presence of MiP fragments (size ranging from 5 to 10 μm, with spherical or irregular shape) has been documented.^31^

Recent studies have shown that the presence of MiP in the trophoblast cytoplasm is associated with decreased cell viability, cell cycle arrest, reduced cell migration and invasion abilities, increased levels of intracellular reactive oxygen species, production of pro-inflammatory cytokines, tumor necrosis factor alpha (TNF-α) and interferon-gamma (IFN-γ) in a dose-dependent manner and important alterations in the epithelial barrier.^32^^,^^33^

## Nervous system

MiP serve as a vector for a wide variety of endocrine disruptors (EDCs) with steroidogenic activity and can bind to different receptors, such as estrogen, androgen, PPAR-γ and AhR. These pathways are part of the complex neuroendocrine regulatory network^34^ that can interfere with hormonal systems, particularly during early life development in the prepubertal period, causing cell and molecular changes in the central nervous system, which can result in behavioral, memory, learning and neurodegenerative problems later in life.^22^

## Digestive system

Generated by several mechanisms and transported through different environmental compartments, MiP can reach the food web and, ultimately, the human body.^35^

The potential risks to human health arising from the unintentional ingestion of NP/MiP are an emerging concern. The presence of MiP in human feces proves that these particles are indeed ingested and pass through the gastrointestinal tract (GIT).^36^

Once consumed, MiP undergoes transformations, which affect the absorption capacity and rates. There are several molecules within the GIT with which MiP can interact, such as proteins, lipids, carbohydrates, nucleic acids, ions, and water. This leads to MiP/NP being encompassed by a collection of proteins known as the “crown”.^37^

Despite being considered chemically inert particles, plastics can adsorb substances, such as additives, heavy metals, proteins, or even microorganisms, on their surface, which can cause greater toxicity. Thus, MiP functions as a Trojan horse, bringing with them a series of environmental contaminants that can interact with the mucus lining the GIT, with epithelial cells, and with the intestinal microbiota, causing cell responses and several physiological changes.^38^

Chemical contaminants can be transferred from mother to child through breastfeeding, depending on its duration and the child's body burden, generally reflecting the maternal exposure burden. Analysis of MiP concentrations in meconium stool samples from babies showed higher levels of MiP than in adults.^36^

Plastic packaging for baby food and baby bottles should be considered potential sources of MiP.^32^^,^^39^ Kadac-Czapska et al. reported that the average number of plastic particles detected in the tested products was 42 ± 27 MiP/100 g, and estimates indicate that infants may be exposed to ingestion of 6 to 176 MiP/day if infant formula is their sole source of nutrition.^40^ Song et al. demonstrated that baby bottles and water bottles release microparticles in quantities ranging from 53 to 393 particles/mL and are influenced by the brand and type of bottle (plastic versus glass).^41^

MiP may have effects on diseases that have not yet been studied, such as food allergy, where they may act by modifying the digestibility of food allergens, increasing intestinal permeability, promoting an inflammatory intestinal environment, or causing intestinal dysbiosis, which may promote sensitization to food allergens^18^ (Table 2).

**Table 2:** Cellular and human health effects associated with exposure to microplastics.^a^^,^^16^^,^^18^, ^19^, ^20^, ^21^

*Immune system*
Increased production of IL-8 by monocytes and macrophages Increased production of IL-6 by monocytes and macrophages Increased production of TNF-α Induction of production of IL-1α, IL-1β, IL-2 and IL-12 Increased histamine release by mast cells and RBL-2H3 cells
*Digestive system*
Cytotoxicity associated with oxidative stress Increased lipid peroxidation Increased reactive oxygen species Increased gastric mucus production Associated with intestinal microbiota dysbiosis
*Nervous system*
Neurotoxicity secondary to the action of pro-inflammatory cytokines at the neuronal level. Some studies suggest a probable association with neurodegenerative diseases.
*Respiratory system*
Association with autophagy secondary to oxidative stress and cytokine-mediated inflammation. Some studies suggest a probable association with the exacerbation of symptoms in chronic respiratory diseases. Alteration of the epithelial barrier associated with increased prevalence and severity of allergic and inflammatory diseases.

a Effects associated with concentration and time of exposure. IL-8, interleukin 8; IL-6, interleukin 6; TNF-α, tumor necrosis factor alpha; IL-1α, interleukin 1 alpha; 1β, interleukin 1 beta; IL-2, interleukin 2; IL-12, interleukin 12; RBL-2H3 cells, cells used in the study of mast cell function.

MiP/NP causes direct intestinal toxicity by acting on the microbiota and indirect toxicity in other organs through the regulation of the gut-brain, gut-liver and gut-lung axes.^42^

In addition to particle toxicity induced by MiP, indirect toxicity caused by the release of additives and monomers is of considerable importance. Plastic additives such as phthalates and bisphenols have been shown to affect adipogenesis and energy balance. Bisphenol A can bind to estrogen receptors and interfere with estrogen signaling to cause obesogenic effects.^43^

## Cutaneous route

Although the cutaneous route is a less efficient route, studies have questioned the possibility of NP/MiP crossing the dermal barrier. The clarification of this issue is essential, not only for individuals with normal barrier function but also, more importantly, for those with compromised skin due to disease (e.g. eczema) or physical abrasion^18^ (Table 1).

The mechanical production method used to manufacture microspheres in beauty and health products, including facial and body scrubs, toothpaste and denture restorations, increases the likelihood of microspheres breaking down into even more harmful MiP.^38^ Ingredients widely used in body lotions, such as urea, glycerol and α‑hydroxy acids, have also increased the ability of MiP to permeate the skin barrier.^44^

## Systemic route

The systemic contact route also occurs predominantly in the hospital environment. Healthcare spending accounts for almost 10 % of the global economy and must continue to grow to provide equitable access to healthcare for the world's growing population. The presence of MiP in the hospital environment (Table 3) is a growing concern due to its potential impacts on human health and the environment.^45^

**Table 3:** Microplastics (MiP) in the hospital environment.^45^, ^46^, ^47^, ^48^

Potential sources
Medical supplies and equipment: syringes, gowns, surgical gloves, intravenous kits and blood bags. Medication containers and packaging.
Medical procedures
Those involving plastic medical devices can release MiP into the hospital environment.
Pharmaceutical products
Some medications/pharmaceutical products may contain MiP as a result of manufacturing and packaging processes.
Medical waste management
Medical waste often includes plastic materials, which are a potential source of MiP if not managed properly.
Environmental impact
The release of MiP in hospital settings can contribute to the general pollution of the environment, including bodies of water and soils.

The healthcare sector consumes enormous quantities of plastics, many of which are unnecessary or overused. After use, if not managed appropriately, plastic waste ends up in landfills or incinerated, practices that allow it to enter the soil and waterways and contribute to air pollution.^46^

Knowing about and attenuating the presence of MiP in the hospital environment requires coordinated action among healthcare professionals, the patient community, industry, policymakers, and the scientific community. Addressing this issue is crucial to protect human health and reduce the environmental impact associated with healthcare.^45^^,^^46^

## Microplastics in the hospital environment

The use of plastics in healthcare is frequent and raises concerns regarding the potential leaching of endocrine-disrupting plasticizers and MiP and NP present in them. The leaching of these particles and MiP from plastic healthcare equipment into the blood of intensive care patients has been associated with several health risks.^45^

Phthalates are a group of phthalic acid esters, non-covalently bound to consumer plastic materials and medical devices to improve their flexibility, softness and extensibility. In humans, they can have toxic effects on the liver, thyroid, kidneys, lungs, reproductive, endocrine, nervous and respiratory systems and are associated with asthma, obesity, autism and diabetes.^46^^,^^47^

Preterm newborns (PTNB) may be exposed to high levels of phthalates during hospitalization in the neonatal intensive care unit (NICU) due to multiple medical procedures to which they are submitted.^47^

A pilot study evaluated the migration of MiP through an infused parenteral nutrition circuit (crystalloid and lipid) using an *ex vivo* experimental setup similar to the clinical administration employed in the NICU of a university hospital.^48^ Intravenous administration of the crystalloid solution, despite being administered through an in-line filter (0.2 μm), allowed 1 to 52 MiP particles (> 25 μm) to be administered over a 72-hour period to a 1-kg neonate. The same solution administered without a filter, respecting the same variables (0.80 MiP/mL at a flow rate of 4.2 mL/h for 72 h), allowed the maximum number of MiP particles to reach 241. With the administration of lipids, the estimated exposure ranged from 8 to 115 MiP particles in a 1-kg neonate in 72 h, despite the presence of an in-line filter (1.2 μm).^48^ According to the authors, the diameter of the observed MiP exceeded the diameter of the pulmonary and tissue capillaries,^46^ allowing their obstruction and subsequent granulomatous microvascular and pulmonary inflammation, similar to that observed with other types of particles.

Thus, exposure to MiP leached from intravenous lines may be an additional and direct route of exposure in humans and neonates in particular. However, to understand the importance of this route of exposure, more energetic actions are needed to reduce the variability of concentrations of released MiP that reach the bloodstream. In-line filters seem to be able to reduce the number of MiP that enter patients intravenously, although no absolute filtration has been observed.^48^

Phthalates leached from various medical supplies and associated exposures were first quantified by Wang & Kannan,^49^ who evaluated 10 types of phthalates, mainly DEHP, released by 72 medical products being used in a NICU: creams/liquids, medical devices, first aid products and intravenous irrigation/infusion fluids.^49^ According to the authors, the total sum of phthalates leached ranged from 0.04 to 54,600 μg; DEHP was found in 99 % of the analyzed samples, with the highest amount leached from respiratory support devices (median: 6560 μg). DEHP was also observed in significant concentrations in products labeled as “DEHP-free”. Direct exposure to phthalates through the use of medical devices and first aid supplies, as well as the dermal application of creams/lotions, were calculated. The highest dose of DEHP exposure of 730 μg/kg body weight/day was determined from the use of an intubation cannula for neonates.^49^

A study of newborns (NB) with idiopathic neonatal hypertension (INH) found that the majority had bronchopulmonary dysplasia (BPD) and common clinical features, regardless of the presence or absence of BPD. This phenotype included low plasma renin activity and a favorable response to treatment with spironolactone. A small prospective study of these patients showed evidence of mineralocorticoid receptor activation due to the inhibition of 11β-HSD2, the enzyme that converts cortisol to the less potent mineralocorticoid, cortisone. Meanwhile, phthalate metabolites have been shown to inhibit 11β-HSD2 in human microsomes.^50^

In general, existing prospective studies on the impact of phthalate exposure on newborns treated in NICUs involve a small number of participants from single centers.^51^^,^^52^ Thus, the manufacture of low-toxicity NICU medical materials for use during critical periods of development could reduce the incidence of BPD in the PTNB population - patient care and public health priority that has eluded neonatal care providers in the current era.^53^

A systematic review evaluated the short- and long-term health effects of phthalate exposure during the neonatal period in PTNB and full-term ones. Phthalate levels were quantified during the neonatal period. Despite the large number of records, only five studies were considered for the review. At two months of corrected age, the sum of DEHP metabolites was associated with worse fine motor coordination, transient alteration of the intestinal microbiota and increased IgM production after vaccination. A positive linear association between the phthalate mixture and the slope of the growth curve has been reported even for PTNB. No relationship was observed between phthalates and BPD.^46^

In contrast, the DINE (Developmental Impact of Neonatal Intensive Care Unit Exposures) cohort, which evaluated 360 PTNB (23 to 33 weeks of gestational age), found that 35 % developed BPD. Exposure to specific phthalate mixtures, especially DEHP, at certain points in PTNB development, was associated with a later diagnosis of BPD in girls at 26 to 30 weeks of the maternal last menstrual period.^53^ Phthalates have been previously identified as being associated with respiratory support equipment.

## Future prospects

The manufacture of medical and disposable equipment without the inclusion of specific types of phthalates is an action to be implemented. If exposure to phthalate mixtures associated with BPD could be reduced or avoided during the susceptible period(s) of development, a reduction in the risk of BPD would be expected. For the highest-risk neonates — those born at less than 29 weeks of gestation and weighing less than 1250 g — a reduction in the incidence and/or severity of the disease could have clinical impacts not only on long-term lung function but also on growth and neurodevelopment.^45^^,^^46^

Given some methodological issues and the scarcity of related studies, further high-quality research is needed to clarify the relationship between early phthalate exposure and health outcomes.

## Conclusions

Unless the plastics value chain is transformed within the next two decades, the risks to the species, marine ecosystems, climate, health, the economy and communities will become unmanageable. However, alongside these risks lie unique opportunities to lead the transition to a more sustainable world.

Because NP/MiP can have multi- or transgenerational effects, it is crucial to develop preventive strategies, promote regulatory measures and minimize exposure to these products as an urgent public health goal.

As members of the pediatric healthcare team, it is crucial that pediatricians be knowledgeable about the impacts of NP/MiP on the studied patients and continue to advocate for safe and healthy environments for the patients.

## Financial support

None declared.

## Conflicts of interest

The authors declare no conflicts of interest.
